# Cytogenetic Screening on Mediterranean Italian River Buffalo Males Intended for Reproduction and Females with Fertility Issues—A Pilot Study

**DOI:** 10.3390/ani15182654

**Published:** 2025-09-10

**Authors:** Angela Perucatti, Francesca Ciotola, Ramona Pistucci, Sara Albarella, Viviana Genualdo, Cristina Rossetti, Roberta Cimmino, Nadia Piscopo, Evaristo Di Napoli, Domenico Incarnato, Orlando Paciello, Vincenzo Peretti, Pietro Parma, Leopoldo Iannuzzi

**Affiliations:** 1 Institute of Animal Production System in Mediterranean Environment (ISPAAM), National Research Council (CNR), 80055 Naples, Italy; angela.perucatti@cnr.it (A.P.); ramona.pistucci@cnr.it (R.P.); viviana.genualdo@cnr.it (V.G.); cristina.rossetti@cnr.it (C.R.); domenico.incarnato@cnr.it (D.I.); 2 Department of Veterinary Medicine and Animal Production, University Federico II, 80138 Naples, Italy; francesca.ciotola@unina.it (F.C.); sara.albarella@unina.it (S.A.); nadia.piscopo@unina.it (N.P.); evaristo.dinapoli@unina.it (E.D.N.); orlando.paciello@unina.it (O.P.); vincenzo.peretti@unina.it (V.P.); 3 National Association of Buffalo Breeders (ANASB), 81100 Caserta, Italy; r.cimmino@anasb.it; 4 Department of Agriculture and Environmental Sciences, University of Milan, 20133 Milan, Italy; pietro.parma@unimi.it

**Keywords:** river buffalo, freemartinism, centromere, cytogenetics, reproduction

## Abstract

In Italy, karyotype analysis is routinely performed on all young males intended for reproduction to screen for chromosome abnormalities. This practice gained prominence following the identification of a complex chromosome abnormality in a well-known bull named Magnifico and its progeny. This procedure significantly enhances the value of the Mediterranean Italian River Buffalo breed, particularly in the context of artificial insemination both within Italy and in other countries importing semen. In the current study, 159 young Mediterranean Italian River Buffalo (MIRB) males intended for reproduction, 30 females with fertility issues, 3 young females and a male co-twin were investigated by analyzing their karyotype using both CBA- and RBA-banding. All males displayed a normal karyotype (2n = 50, XY), although one animal displayed an abnormal X-chromosome with C-band polymorphism, as it is C-band negative when this chromosome normally shows the largest C-band among all chromosomes. This result was also confirmed by the FISH-mapping technique using three bovine BAC-clones containing SAT-I, SAT-III and SAT-IV. Cytogenetic investigation showed that seven females, along with one male co-twin, were affected by freemartinism (XX/XY leukocyte chimerism) and were subsequently removed from the farm. In two of these females, clinical and necroscopic examinations showed severe damage of the internal reproductive organs.

## Simple Summary

A total of 159 Mediterranean Italian River Buffalo young males intended for reproduction, 30 females with fertility issues, 3 three young females and a male co-twin were investigated by analyzing their karyotype using both CBA- and RBA-banding techniques. All males displayed a normal karyotype (2n = 50, XY), although one animal displayed an abnormal X-chromosome with C-band polymorphism, as it is C-band negative, when this chromosome normally shows the largest C-band among all chromosomes. This result was also confirmed by the FISH-mapping technique using three bovine BAC-clones containing SAT-I, SAT-III and SAT-IV. Cytogenetic investigation in females showed that seven females, along with one male co-twin, were affected by freemartinism (XX/XY leukocyte chimerism) and were subsequently removed from the farm. Clinical and necroscopic examinations in two females showed severe damage to the internal sex organs.

## 1. Introduction

In Italy, as part of the genetic control conducted by the National Buffalo Breeder Association (ANASB), karyotype analysis is routinely performed on all young males intended for reproduction to screen for chromosome abnormalities [1]. This practice gained prominence following the identification of a complex chromosome abnormality in a well-known bull named Magnifico and its progeny [2]. This procedure significantly enhances the value of the Mediterranean Italian River Buffalo (MIRB) breed, particularly in the context of artificial insemination both within Italy and in other countries importing semen.

Cytogenetic investigations have also been performed in female river buffaloes with fertility issues (lack of estrus and pregnancy also in the presence of the bull, low number of calves produced during the reproductive age). The studies undertaken of these females showed the presence of chromosome abnormalities, mostly involving sex chromosomes, in about the 20% of examined females with fertility issues (reviewed in [1]). Sex chromosome abnormalities were related to freemartinism (XX/XY leukocyte chimerism) in most of cases, but in some cases X-monosomy, X-trisomy and sex reversal syndrome (XY females) were found (reviewed in [1]).

Chromosome abnormalities involving the autosomes seem to be rare in river buffaloes. Indeed, few of these abnormalities have been reported so far (reviewed in [1]). Some cases of complex chromosome abnormalities involving the autosomes were found in a river buffalo female with reduced fertility and her son [3] and, two years later, in Magnifico, a well-known bull [2]. These chromosome abnormalities occurred with a fission of the bi-armed river buffalo chromosome 1 (BBU1), followed by centric fusion of BBU1p with BBU23 in the female and her son [3], and with BBU18 in the bull and part of his progeny [2]. After this discovery, the Italian National Buffalo Breeder Association (ANASB) started routine cytogenetic analyses for all males intended for reproduction. Part of these males were cytogenetically investigated within the GENOBU research project and in the current study we report the results of this investigation. The results obtained displayed that the karyotype of MIRB males investigated so far is largely normal, since only one individual with an abnormal X-chromosome has been identified. In addition, we investigated thirty females with fertility issues, three young females and a male co-twin all belonging to the MIRB breed. These last four cases were examined at the request of the breeders, as they were born from twin births.

## 2. Materials and Methods

### 2.1. Animals

One hundred fifty-nine young Mediterranean Italian River Buffalo (MIRB) males destined for reproduction from 20 different farms in the provinces of Salerno and Caserta (Campania region, Southern Italy) were investigated along with thirty females displaying fertility issues, three young females and a male co-twin and are all MIRB animals raised in the same provinces. Reproductive problems in females included failure to conceive despite repeated exposure to a bull, and a low number of calves produced during their reproductive life.

### 2.2. Cytogenetics Analyses

Peripheral blood samples were collected and, on the same day, delivered to the cytogenetic labs of both CNR-ISPAAM (Portici, Naples) and the Department of Veterinary Medicine and Animal Production (Naples). Two types of cell cultures were conducted: (A) normal cultures addressed to study the diploid number and frequency of male and female cells using the CBA-banding technique and (B) cultures treated for late BrdU-incorporation to obtain R-banding patterns useful for the karyotype construction and possible application in the FISH-mapping technique.

Whole blood (1 mL) was cultured for 72 h at 37.5 °C in 10 mL of RPMI-medium enriched with fetal calf serum (10%), antibiotic and mycotic solutions (1%) and Concanavalin A (1.5%) as mitogen. For cell cultures B, BrdU (10 µg/mL) and Hoechst-33258 (20 µg/mL) were added 6 h before cell harvesting to obtain enhanced R-banding patterns. One h before harvesting, Colcemid (0.1 µg/mL) was added for both cell cultures A and B.

After hypotonic treatment cells were fixed three times with acetic acid/methanol (1:3). Finally, cells were spread on slides to be ready for banding treatments.

### 2.3. CBA-Banding

Slides obtained for “normal cell cultures A” and aged at least one week at the room temperature were treated for the CBA-banding technique, as earlier reported [4]. Indeed, the C-banding technique allows for the easy identification of sex chromosomes (the X-chromosome shows the largest C-band among all acrocentric chromosomes with a proximal additional C-band positive, while the Y-chromosomes appears entirely heterochromatic or with a distal-telomeric positive C-band, depending to the degree of chromosome denaturation). At least 100 cells for each animal were recorded and later studied to establish the exact diploid number, percentage of XX and XY cells and correct C-banding pattern. In the male co-twin only 30 cells were examined due to poor cell growth.

### 2.4. RBA-Banding

Slides obtained from “cell cultures B” followed the protocol reported in [4]. First, slides were stained with Hoechst-33258 for 10 min, then washed with distilled water, mounted with a coverslip and finally exposed to UV-light for 30 min. Slides were then washed with distilled water, air-dried and finally stained with acridine orange (0.1% in P-buffer) for 10 min. Then, the slides were washed with both tap and distilled water and air dried. Slides were then mounted with a cover slip in P-buffer and observed under a fluorescence microscope. At least 30 cells were recorded for each animal on the basis of good quality of R-banding. At least two karyotypes for each animal were generated following the river buffalo standard karyotype [5].

### 2.5. FISH-Mapping

One male with an abnormal X-chromosome with C-band polymorphism and a control male with normal karyotype were further investigated through the FISH-technique using three BAC-clones containing satellite DNA (SAT-I, SAT-III and SAT-IV) [6]. Detection of the hybridization signal was as previously published [7]. Slides with fresh cell suspension from R-banding treated cells, as reported above, were incubated all night at 50 °C and then treated for the dual color FISH-technique to obtain simultaneous double signals using two different BAC-clones and microscope filter combinations. After hybridization and washing of the slides, digoxigenin-labeled chromosomes were detected with Rhodamine (RODH) conjugated to anti-digoxigenin (Roche, Basel, Switzerland) and biotin-labeled probes were detected with FITC-conjugated to avidin (Vector Laboratories, Newark, CA, USA). Chromosomes were counterstained with DAPI and mounted in Vectashield (Vector Laboratories). Two images with double fluorescent signals of the same metaphase were acquired using a Leica cytogenetic workstation equipped with a fluorescence microscope and then superimposed to each other to obtain a single image with two hybridization signals (red and green) on chromosome centromeric regions.

### 2.6. Gynecological and Histopathological Examination

Two females (Cases B2250 and B202) underwent gynecological examination, including rectal palpation and ultra-sonographic analysis. In Case B2250, after slaughter, the reproductive tract was collected and submitted to gross and histopathological examination in the necropsy room of the Department of Veterinary Medicine and Animal Production using standard necropsy protocol [8,9,10]. Tissue samples for histological analysis were collected from the blind-ended cylindrical structures, urinary bladder, bladder protrusion, bladder neck and ovary. Samples were fixed in 10% buffered formalin for 24 h and then embedded in paraffin. For histological examination, 3–5 μm sections were stained with hematoxylin and eosin. Slides were then scanned, observed and photographed using the Pannoramic II scanner (3DHISTECH, The Digital Pathology Company, Öv utca 3, 1141 Budapest, Hungary).

## 3. Results

### 3.1. Young MIRB Males

**Figure 1:**
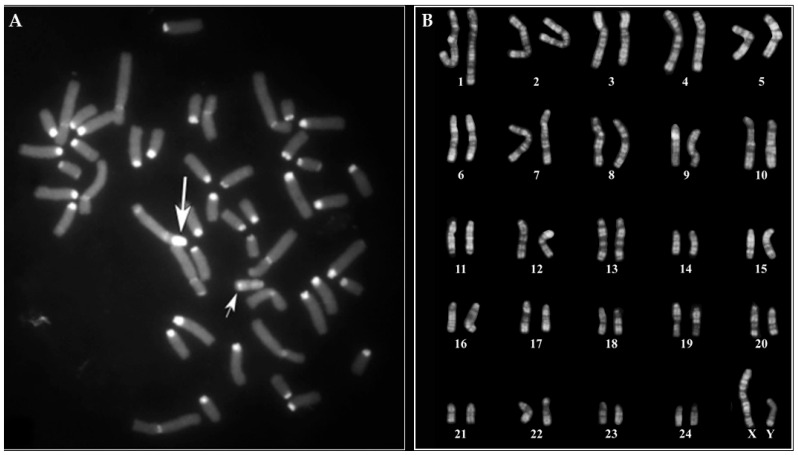
(**A**) CBA-banding in a single metaphase spread from one of the 159 young MIRB males destinated for reproduction. X- (large arrow) and Y- (small arrow) chromosomes are indicated. Note the prominent C-band in the X-chromosome, the largest one among all chromosomes and the distal positive C-band in the Y-chromosome. (**B**) Normal RBA-banded karyotype from a single cell of another young MIRB male (among the 159 destined for reproduction), representative of the group.

All males analyzed presented a normal karyotype (2n = 50, XY) according to both CBA- (Figure 1A) and RBA-banding (Figure 1B) techniques.

**Figure 2:**
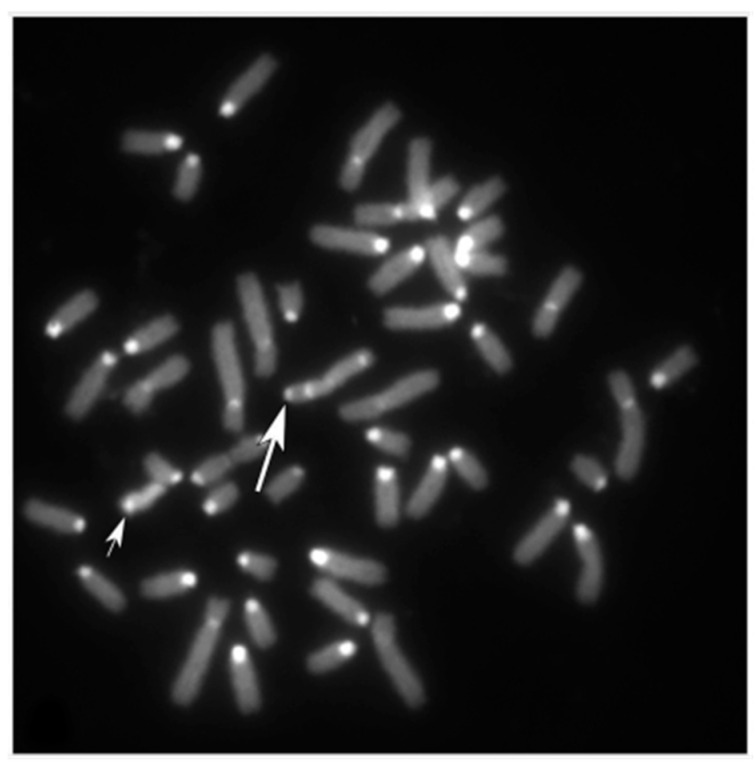
CBA-banding in metaphase spread from a MIRB male carrying an abnormal X-chromosome (large arrow) carrying C-band polymorphism with only a very small positive region at the centromeric tip. Small arrow indicates the Y-chromosome (note the positive C-band distally located).

Only one male showed an unusual X-chromosome as revealed by CBA-banding (Figure 2).

**Figure 3:**
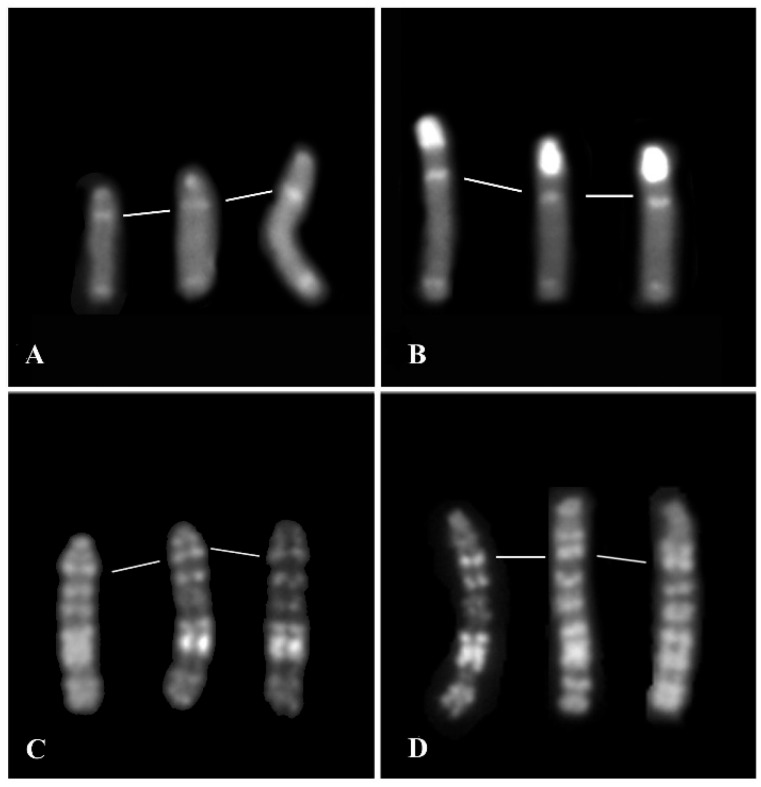
Comparison between abnormal (**A**–**C**) and normal (**B**–**D**) river buffalo X-chromosomes by using CBA- (**A**,**B**) and RBA- (**C**,**D**) banding techniques from different cells. The lines indicate the homologous and most conservative proximal chromosome band in both banding techniques. Note the different size of the centromeric regions between the two types of Xs and the prominent and large C-band in the normal X compared with the abnormal one.

Indeed, the X was C-banding negative (with a very small positive area at the centromeric tip) (Figure 3A) when compared with the normal X which shows a prominent C-band, the largest one among all river buffalo chromosomes (Figure 1A and Figure 3B). The centromeric region of the abnormal X-chromosome (C-band polymorphism) appears much smaller than a normal one, also when comparing abnormal and normal X by the RBA-banding technique (Figure 3C and D, respectively).

**Figure 4:**
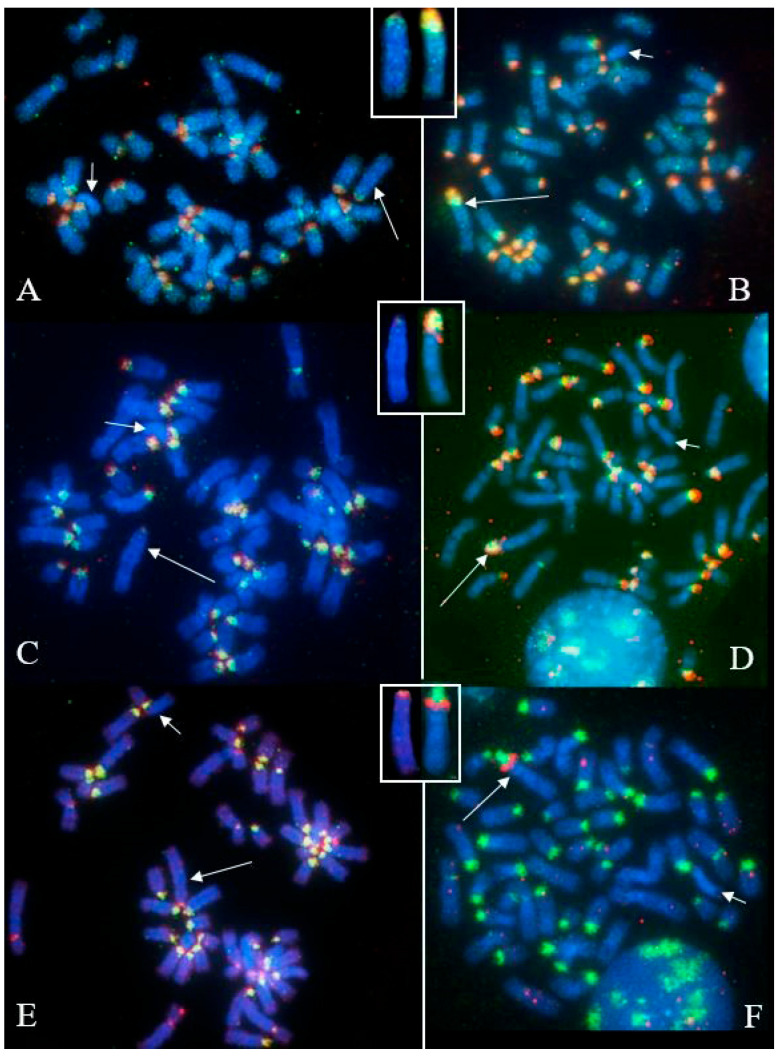
Dual color FISH in the MIRB male metaphases with an abnormal chromosome X (**A**,**C**,**E**) and control normal male (**B**,**D**,**F**) with SAT-I (red signals) and SAT-III (green signals) (**A**,**B**), SAT-I (red signals) and SAT-IV (green signals) (**C**,**D**), SAT-III (red signals) and SAT-IV (green signals) (**E**,**F**). Enlarged details of the X-chromosomes are reported between metaphases to better show the difference in signals between the abnormal (left) and normal (right) X-chromosomes. Note the presence of very small signals at the centromeric tips only of the abnormal X (**A**,**C**,**E**), compared with the normal one (**B**,**D**,**F**). Large and small arrows indicate X- and Y-chromosomes, respectively. Note also that no hybridization signals were detected on the Y-chromosome.

This abnormal C-banding X-chromosome was also confirmed when using the FISH-technique with three SATs (Figure 4). Indeed, all three SATs showed very small hybridization signals in the abnormal X (tip of the centromere) when compared with the normal one (Figure 4).

This is the first time that a similar C-band polymorphism in river buffalo has been reported. Although this subject appeared to have a normal phenotype, the breeder quickly removed it from the farm; thus we were unable to monitor it in order to determine if the anomalous X was a de novo instance or inherited from his parents. We were unable to investigate his parents or relatives.

### 3.2. Females with Fertility Issues

**Figure 5:**
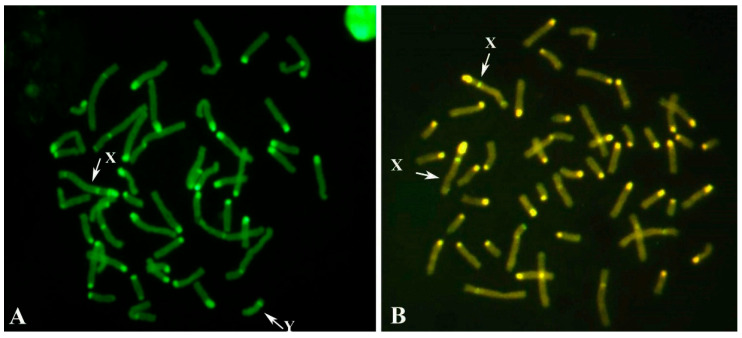
CBA-banding in male (**A**) and female (**B**) cells of female river buffalo freemartin (XY/XX leukocyte chimerism). Note the distal C-band positive in the Y-chromosome.

Among the thirty MIRB females with reproductive problems, four were identified as freemartins, exhibiting XX/XY leukocyte chimerism (Figure 5). In addition, three young females and one male co-twin were also diagnosed with freemartinism, bringing the total number of cytogenetically confirmed cases to eight.

Table 1 reports the percentage of male and female cells in these eight freemartin individuals. The proportion of XY (male) cells ranged from 0.9% to 90%. In the male co-twin, only three XY cells were detected among the 30 metaphases analyzed; however, the presence of the Y-chromosome was also confirmed by PCR testing performed on whole blood extracted DNA to verify the presence of SRY and AMELY genes.

Two freemartin females (cases B2250 and B202) underwent more in-depth investigation, including clinical assessment and, in the Case B2250, post-mortem histopathological examination was also performed after slaughter.

#### 3.2.1. Case B2250

**Figure 6:**
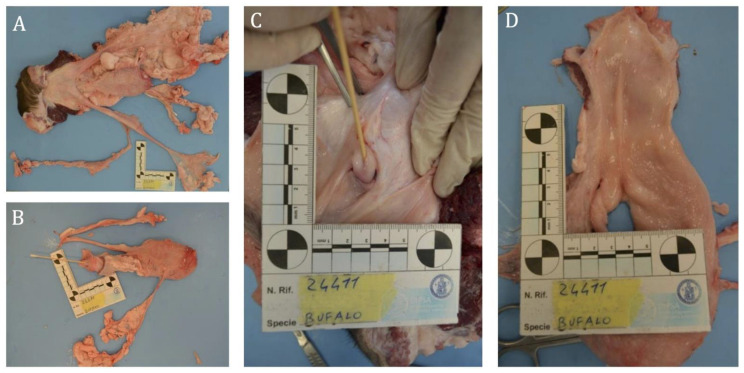
Case B2250: gross appearance of a female buffalo’s reproductive system (**A**–**D**). Four elongated, cylindrical, blind-ended and without lumen structures protrude from the ventral bladder wall (**A**). Distal to the bladder neck, a 2.5 cm-long mucosal invagination is visible. At its distal end, a protuberance with a one cm-wide base and a sharply tapered, acute-angled apex is observed, featuring an opening into the bladder lumen (**B**–**D**).

At the gynecological examination, the female showed a normal vulva but an immature reproductive apparatus, hypertrophic cervices and non-identifiable ovaries by echography. The histopathological examination revealed the following findings: the urinary bladder appeared normally structured, four blind-ended cylindrical structures were observed embedded in its ventral wall, lacking a macroscopically visible lumen and interpreted as embryonic ducts. Just distal to the bladder neck, a mucosal invagination approximately 2.5 cm long was noted, ending in a protrusion about one cm wide, which communicated with the bladder lumen via a canalized duct (Figure 6).

**Figure 7:**
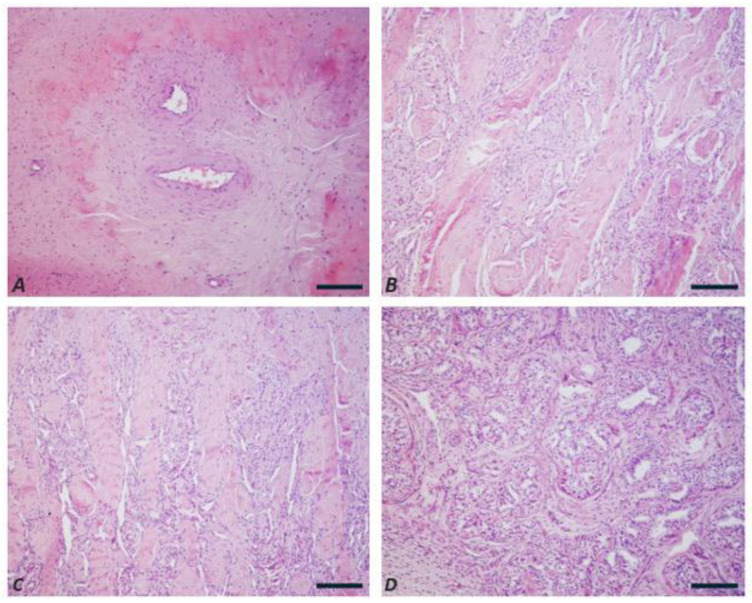
Case B2250: morphological findings of the female buffalo’s reproductive system (**A**–**D**). Representative hematoxylin and eosin staining of blind-ended cylindrical structures (**A**), bladder protrusion (**B**,**C**) and ovary (**D**). (**A**) The blind-ended cylindrical structures in the cut section were compatible with endothelium-lined blood vessels enclosed in an embryonic connective material defined in shape by stellate mesenchymal cells enclosed in an amorphous, eosinophilic material. (**B**,**C**) The bladder protrusion showed underneath a multistratified, normo-structured epithelium, a layer of connective tissue enclosing trabeculae consisting of collagen fibers and smooth muscle organized to form sponge-like structures. The lumen of the trabeculae appears covered by endothelial cells (*corpora cavernosa*). (**D**) The ovary sections showed the presence of cylindrical cells organized in a tubular pattern and separated by thick connective tissue septa. Original magnification, 200×. Scale bars, 20 µm.

An ovoid structure (~0.5 × 1.5 cm), consistent with an ovary, was found embedded in the surrounding fat. Histologically, the blind-ended structures contained endothelial-lined vessels embedded in embryonic connective tissue with stellate mesenchymal cells. The bladder protrusion showed trabeculae of collagen and smooth muscle resembling cavernous bodies. A duct lined by transitional epithelium was observed at the bladder neck. The ovarian tissue showed tubular structures composed of columnar cells with moderately eosinophilic cytoplasm, paracentral nuclei, prominent nucleoli which were separated by thick connective septa (Figure 7).

#### 3.2.2. Case B202

The female, upon gynecological examination, displayed absence of the uterus and ovaries, hypo-plastic vulva and rudimentary uterine structures at the level of the cervix. Phenotypically, the animal exhibited marked male characteristics, including large horns, a prominent rump and a highly muscular forequarter.

## 4. Discussion

Clinical cytogenetics relating to domestic animals originated practically when cattle rob (1;29) was discovered in Swedish red cattle [11] and demonstrated harmful effects on reproduction [12,13] due the production of unbalanced gametes (and subsequent embryos) in the carriers, especially in females. Many cytogenetic labs were created in various countries, and this translocation has been found in more than 50 different breeds [14], especially from beef and some local breeds. This translocation apparently originated from a simple centric fusion translocation between chromosomes 1 and 29 but a series of studies demonstrated that rob (1;29) originated from complex chromosome rearrangements (reviewed in [15]). Rob (1;29) can also be and easily detected by simple qPCR [16].

Chromosome abnormalities in river buffalo have only been addressed in the past in females with fertility issues. About 20% of females with fertility issues are carriers of sex chromosome abnormalities (reviewed in [1]). Most of these females were found to be freemartin (XX/XY leukocyte chimerism), a few cases were found affected by of X-monosomy, X-trisomy and sex reversal syndrome (reviewed in [1]).

Only one female and her son were found to be affected by a complex chromosome abnormality involving autosomes. Indeed, a centric fission of the bi-armed BBU1 originated two acrocentric chromosomes BBU1q and BBU1p, the former remaining acrocentric, the latter was fused by centric fusion translocation with BBU23 [3]. This female displayed a reduced fertility (two calves in five years during her reproductive life) and one of her sons was affected by the same chromosome abnormality. The same type of chromosome abnormality was found two years later in a very extensively used reproduction bull (named Magnifico) and part of his progeny [2]. Indeed, in this bull fission also occurred in the bi-armed BBU1 with BBU1q remaining acrocentric but with BBU1p fused by centric fusion translocation with BBU18 [2]. However, a sperm-FISH analysis on the bull carrier showed a very low percentage of unbalanced sperm [17]. This observation coincides with what has been observed in cattle, where alternating segregation of gametes helps prevent the formation of unbalanced gametes [18].

After this discovery, the Italian Buffalo Breeder Association (ANASB) started cytogenetic screening all males intended for reproduction, as we conducted in the current study.

In this study, the cytogenetic investigation performed in MIRB males demonstrated that all individuals showed a normal karyotype (2n = 50, XY) given an additional genetic value to the river buffalo male reproducers in light of the large use of their semen, especially from selected animals with high EBV (Estimated Breeding Value), both in Italy and foreign countries. Only one male (2n = 50, XY) showed an abnormal X, being C-band negative with a very small positive band at the centromeric tip (Figure 2 and Figure 3A), when BBU-X normally shows the largest and strongest C-band in river buffalo karyotypes (Figure 1A, Figure 3B and Figure 5). This result was also confirmed by using specific probes mapping the chromosome centromeric regions (SAT-I, SAT-III and SAT-IV) (Figure 4). This is the first case even found in this species and no other cases have been found in other bovid species showing a similar acrocentric X-chromosome C-band positive (Eland type), the most common X-chromosomes in the *Bovidae* family are probably very similar to the ancestral *Bovidae* X-chromosome [19]. The male carrier was apparently phenotypically normal even if we were unable to follow this animal due to fast suppression by the breeder. Moreover, we could not investigate both parents and relatives to verify if the abnormal X was inherited from the parents or if it was a de novo case. At the moment, it appears difficult to establish the possible effects of this type of chromosome abnormality on fertility, being the first case found in this species. Possible problems could originate during cell mitosis (and subsequent meiosis) when chromosomes are all attached to the cellular spindle along their centromeric regions, although the presence of a very small HC-region at the X-centromeric tip, as shown by both C-banding and FISH-mapping, could ensure the correct chromosome cell division. Further studies on similar cases are necessary to better investigate this new atypical X-chromosome. Certainly the continuation cytogenetic screening of males intended for reproduction, as recommended by the Italian National Buffalo Breeders Association (ANASB), represents the best way to select males without chromosome abnormalities which can be easily spread in their progeny, especially when using AI, as occurred with the famous MIRB-bull Magnifico [2].

The present study confirmed the occurrence of freemartinism in river buffaloes, identifying eight cases through cytogenetic and/or molecular analyses performed on thirty MIRB females with fertility issues and three young MIRB females (Table 1) suspected of being freemartins. All three young females were born from twin pregnancies: one with a living male co-twin and two whose male co-twins died at or shortly after birth. These animals were analyzed between one to three months of age following the breeders’ request for early cytogenetic testing to decide whether to retain or cull them from the herd. Interestingly, in one of these confirmed cases (a male co-twin) only a few XY cells were detected among the 30 metaphases analyzed, but the presence of the Y-chromosome was also confirmed by PCR.

This finding highlights the importance of analyzing a higher number of metaphases—at least 50 per animal—to reduce the risk of false negatives when screening for XX/XY leukocyte chimerism. Although this may suggest that molecular analysis alone is sufficient for diagnosing freemartinism, conventional karyotyping remains a fundamental tool, as it allows for the detection of additional structural chromosomal abnormalities that may not be identified by molecular methods alone.

Two of the freemartin females underwent detailed clinical evaluation by a veterinary practitioner, revealing evident abnormalities in internal genitalia, such as the absence of ovaries and the presence of rudimentary uterine structures. In one of these females, pronounced male secondary sexual characteristics were also observed (e.g., large horns and prominent withers), a finding consistent with previous reports in the literature (reviewed in [1]). This is an important feature because most freemartin females are born as a single co-twin, as occurred in at least four of the eight freemartin cases found in the present study, with the male co-twin dying during the early embryonic life and being absorbed. Often the breeders cannot know that a female is freemartin and keep it in the farm as a normal female. Only later, when the female reaches reproductive age and does not remain pregnant, the breeder tries to understand the reason, asking for the veterinary and subsequent cytogenetic investigation. The identification of male traits in these females at a young age could anticipate the diagnosis of this syndrome by several months (in some studied cases, also several years), saving time and a lot of money as the females are generally sterile, as clearly demonstrated in all female river buffalo found freemartin so far (reviewed in [1]) and in the present study, and demonstrated in Figure 7 where serious damage was found in the freemartin female.

In the past we tried to correlate the percentage of male cells in freemartin females with the type of damage produced by the XY-cells. We reached the conclusion that the time of XY cell (and hormone) transfer between the two twins of the opposite sex is more important than their percentage. It should be noted that damage to sexual development in female co-twins derives from the fact that not only cells (XY) from the male co-twin are transferred via the anastomoses, but also hormones produced by the developing testicle, which masculinize the female, who begins sexual development at least one week after the male [20,21].

Freemartin syndrome is essentially related to the frequency of twinning, which is significantly higher in cattle, ranging from 2 to 4% in dairy breeds and reaching up to 6% in older cows [22] compared to river buffalo, where the reported twinning rate is only 0.14% [23]. However, most freemartin females in river buffalo are single born (reviewed in [1]) and confirmed by our findings (Table 1). This suggests that the actual incidence of twinning in Mediterranean Italian River Buffalo, at least during early embryonic development, may be much higher than what is typically observed at birth.

## 5. Conclusions

Cytogenetic screening of animals in livestock production is essential for preserving the genetic heritage of the species. The river buffalo is no exception, and since 2013, all males used for breeding have undergone this screening.

This study presents the results of cytogenetic screening on 193 subjects of both sexes. The results displayed that eight subjects were carriers of freemartinism with severe damages to internal sex organs and one male was, for the first time, the carrier of a non-conforming X-chromosome.

The results confirm the importance of early screening, especially with regard to sex chromosome abnormalities, particularly regarding freemartinism, which often does not cause external phenotypic alterations in females.

## Figures and Tables

**Table 1:** Age and percentage of male (M) and female (F) cells in the eight animals found freemartin.

Case	Age ^1^	Cells ^2^	Sex	% XX	% XY	C.I. 95% XY Cells ^3^
B2250	32	100	F	89.1	9.9	4.1–15.7
B202	26	100	F	51.0	49.0	39.2–58.8
B2019 *	3	30	M	99.1	0.9	2.5–4.3
B2020 *	3	100	F	95.8	4.2	0.3–8.1
B1650	3	100	F	86.2	13.8	7.0–20.6
B577	22	100	F	68.0	32.0	22.9–41.1
B475	1	100	F	89.0	11.0	4.9–17.1
B179	30	100	F	10.0	90.0	84.1–95.9

^1^: age in months. ^2^: number of observed cells (at least). ^3^: 95% confidence interval of the XY cells population percentage. *: co-twins.

## Data Availability

Dataset available on request from the authors. The raw data supporting the conclusions of this article will be made available by the authors on request.

## Abbreviations

The following abbreviations are used in this manuscript:

CBA	C-banding by Acridine Orange Staining
RBA	R-banding by late BrdU-incorporation and Acridine Orange Staining
BAC	Bacterial Artificial Chromosome
FISH	Fluorescence In Situ Hybridization
SAT	Satellite DNA
BBU	River Buffalo Chromosome (*Bubalus bubalis*)
BrdU	5-bromo-2′-deoxyuridine
FITC	Fluorescein Isothiocyanate
RODH	Rhodamine
DAPI	4′,6-diamidin-2-fenilindol
